# Case Report: Immune reconstitution inflammatory syndrome after hematopoietic stem cell transplantation for severe combined immunodeficiency

**DOI:** 10.3389/fimmu.2022.960749

**Published:** 2022-09-26

**Authors:** Shuangjun Liu, Feng Huo, Guorui Dai, Jie Wu, Maoquan Qin, Huawei Mao, Quan Wang

**Affiliations:** Beijing Children’s Hospital, Capital Medical University, Beijing, China

**Keywords:** severe combined immunodeficiency, HSCT = hematopoietic stem cell transplant, BCG disseminated tuberculosis, IL2RG, IRIS - immune reconstitution inflammatory syndrome

## Abstract

We report a case of immune reconstitution inflammatory syndrome (IRIS) after hematopoietic stem cell transplantation (HSCT). The patient had sever bacillus Calmette–Guerin (BCG) vaccine–caused disseminated infection and had received allogeneic HSCT for X-linked severe combined immunodeficiency disease. After HSCT, complicated by treatment-responding veno-occlusive disease and acute graft-versus-host disease, at the time when immunosuppressants were withdrawn, the patient experienced recurrent fever accompanied by elevated inflammatory indicators. After receiving glucocorticoids and ibuprofen, the patient’s condition improved, and a diagnosis with BCG-related IRIS was made.

## Introduction

Severe combined immunodeficiency (SCID) is a life-threatening syndrome characterized by severe dysfunction of cellular and humoral immunity owing to impaired T-cell and B-cell development or function. Interleukin-2-γ chain receptor (IL2RG) mutation is the most common cause of SCID. Although gene therapy is effective for some forms of SCID, hematopoietic stem cell transplantation (HSCT) is the treatment of choice. Immune reconstitution inflammatory syndrome (IRIS) is primarily defined as the deterioration of the clinical manifestations of a pre-existing infection that occurs in patients with acquired immune deficiency syndrome who initiate anti-retroviral therapy as their immune function improves (1). We report a case patient who received the live bacillus Calmtte-Guerin (BCG) vaccine at birth, was subsequently diagnosed with SCID, and who developed post-HSCT IRIS related to disseminated BCG-associated infection.

## Case description

A 4-month-old male infant with non-consanguineous healthy parents received BCG vaccination at birth, after which vaccination, scattered pustules appeared on his trunk and limbs. At the age of 3 months, some of the rash scabbed followed by development of recurrent low to moderate fever. He was transferred to our hospital (a tertiary teaching children’s hospital).

Laboratory investigation revealed remarkably T-cell and natural killer cell (NK cell)-cell counts of the patient indicating a diagnosis of (T-B+NK−) severe combined immunodeficiency (SCID). Further examination including whole exome sequencing, blood culture, skin biopsy, next-generation sequencing (NGS), and computed tomography confirmed that the patient had a *de novo* mutation of IL2RG (IL2RG c.139_148delACTGACTCCC hemizygote) with disseminated infection of BCG strain of *Mycobacterium bovis* which affected the spleen, liver, kidney, bone, and subcutaneous tissue.

Before HSCT, he received anti-tuberculous treatment including linezolid, meropenem, isoniazid, rifampicin, ethambutol, and clarithromycin. However, he still suffered from high fever and developed complications such as dyspnea requiring mechanical ventilation, multiple-organ dysfunction, hypersplenism, disseminated intravascular coagulation, secondary hemophagocytic syndrome, and septic shock. Considering the patient’s critical condition, we performed a non-conditioned allogeneic HSCT in ICU (non-laminar flow ward), by infusion of bone marrow and peripheral blood stem from his HLA-matched (10/10) sibling without graft-versus-host disease (GVHD) prophylaxis.

**Figure 1:**
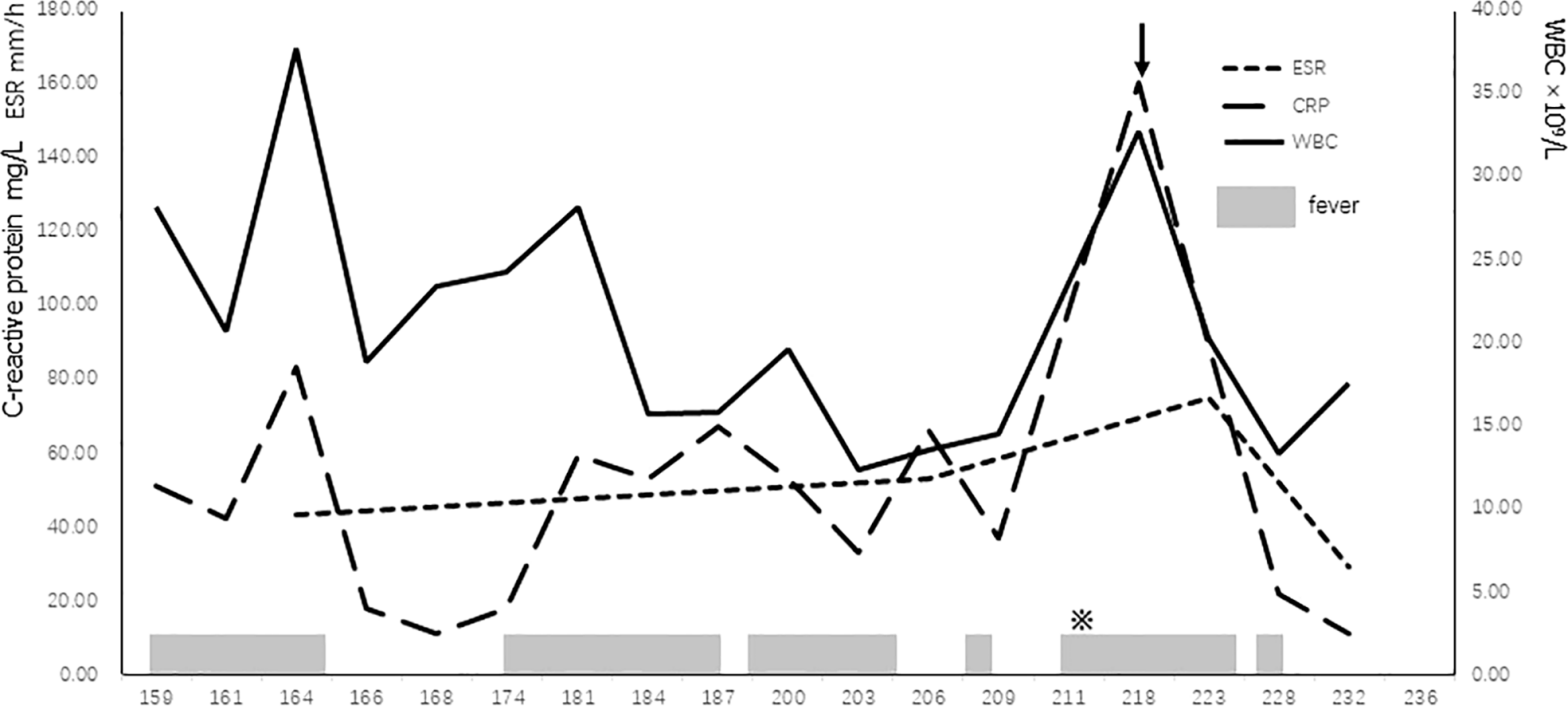
Trajectory of patient’s WBC and inflammatory markers with clinical events. ※, more subcutaneous nodules; arrow, administration of methylprednisolone; ESR, erythrocyte sedimentation rate; WBC, white blood cell.

On days +4 and +11 after HSCT, respectively, the patient developed hepatic veno-occlusive disease (VOD) and acute GVHD—both improved after defibrotide, methylprednisolone, and cyclosporine A (CSA) treatment. On day +14, donor cells accounted for 59.03%, indicating mixed chimerism. However, the spleen infection was worsening and painless subcutaneous nodules appeared on the trunk and limbs of the patient on day +36. On day +47, an ultrasound-guided splenic abscess puncture was performed, staining for acid-fast bacilli was positive, and NGS showed BCG strain of *Mycobacterium bovis*, although the pus culture was negative. As monitoring showed a fall of donor chimerism to 38.33%, he received another infusion of donor hematopoietic stem cells from the donor on day +89. Because of the poor implantation of B cells, he received regular supplementation with intravenous immunoglobulins (IVIG). The patient gradually improved and was discharged on day +126 after HSCT with combined anti-BCG treatment (clarithromycin, ethambutol, isoniazid, linezolid, and rifampicin) and CSA (15 mg/day). During the follow-up, it was realized that the parents mistakenly gave higher dose of CSA (100 mg/day), and although CSA plasma concentration was normal (49.3 µg/L), it was decided to be stopped on day +151. Following this, from days +158 to +195, the patient developed intermittent fever accompanied by non-specific symptoms such as poor appetite and fatigue. Although blood cultures remained negative, he received treatment with imipenem cilastatin and cefoperazone sulbactam sodium, resulting in temporarily normalization of his temperature. However, from day +211, prolonged fever reappeared, the number of painless subcutaneous nodules increased, but his liver and spleen lesions were reduced. Only Torque teno virus was detected in the peripheral blood NGS, whereas urine analysis, stool examination, *Clostridium* difficile toxin, rotavirus, serum Epstein-Barr virus (EBV)-DNA, and cytomegalovirus (CMV)-DNA were all negative. During this period, the results of inflammatory markers [C-reactive protein (CRP), erythrocyte sedimentation rate (ESR), and leukocytes) fluctuated: CRP between 11 and 160 mg/L, leukocytes from 13.28 to 31.25 × 10^9^/L, ESR between 43 and 75mm/h, whereas procalcitonin remained normal. IL-1β, IL-5, IL-6, IL-8, IL-17, and interferon-γ were all increased, especially IL-6 (99.51 pg/ml). CD4+ cells increased from 1005 cells/µl (day +122) to 1,671 cells/µl (day +209) (**Figure 1**).

The patient was diagnosed with IRIS on the following basis: i) no accompanying symptoms except fever; ii) no deterioration of the previous *M. bovis* BCG strain infection; and iii) no potential etiological evidence related to clinical symptoms (2).

On day +218, methylprednisolone (1.2 mg/kg/day) was administrated with no effect. Hence, on day +224, the dose was increased (to 1.8 mg/kg/day) and combined with ibuprofen (20 mg/kg/day, Q8H). As temperature returned to normal the next day, the dose of methylprednisolone was gradually reduced, and methylprednisolone and ibuprofen were stopped after 3 and 2 weeks, respectively. On day +255, fever returned but normalized following the planned monthly IVIG (400 mg/kg, 5 g). However, about half a month later, when fever developed again, the schedule of administration of 5 g of IVIG every 4 weeks was changed to 2.5 g of IVIG every 2 weeks. Since then (1 year and 4 months after HSCT), the patient has no further fever, and significant progress has been observed in his intellectual and physical development.

Clarithromycin and linezolid were discontinued on day +326, and isoniazid and rifampicin are still continued as anti-BCG treatment. The most recent chimerism and CD4 + cell count on day + 479 were 54.39% and 4,590 cells/µl, respectively (chimerism, lymphocyte count, and anti-BCG treatment are shown in **Table 1**).

**Table 1:** List of major episodes.

Post-HSCT	Chimerism	ALC (×10^9^/L)	CD3%	CD19%	Follow-up	Treatment modification
−29		0.16	3.4	90.7	Disseminated infection with BCG strain of *Mycobacterium bovis*	I + R + L + EB
4		0.3			VOD	I + EB + C + amikacin + levofloxacin Defibrotide
11	59.03	15.13	83.4	3	GVHD	CSA
89	38.33	4.25	77.1	4.2		Re-injection of donor hematopoietic stem cells
126						Discharge
151		14.65	74.2	4.5	CSA concentration 49.3 µg/L	Cessation of CSA I + R + L + EB + C
158		12.08			Fever	I + R + L + C imipenem cilastatin
164		12.31			Transient diarrhea	I + R + L + C imipenem cilastatin
180	65.22	15.45			Fever	I + R + L + C cefoperazone sulbactam sodium, IVIG
195		11.61			Fever	I + R + L + C
211		7.28	85.5	4.7	Fever and increase of painless subcutaneous nodules	I + R + L + C IRIS
218		7.33			Fever	Methylprednisolone at 1.2 mg/kg/day I + R + L + C
224		7.26			Fever	Ibuprofen at 20 mg/kg/day, methylprednisolone at 1.8 mg/kg/day I + R + L + C
225		3.61			Normal temperature	Ibuprofen for 2 weeks and methylprednisolone for 3 weeks I + R + L + C
255					Fever and increase of painless subcutaneous nodules	IVIG I + R + L + C
325						I + R
479	54.39	21.1	55	36.7		I + R

ALC, absolute lymphocyte count; C, clarithromycin; CSA, cyclosporine A; EB, ethambutol; GVHD, graft-versus-host disease; IVIG, intravenous immunoglobulin; I, isoniazid; L, linezolid; R, rifampicin; VOD, hepatic veno-occlusive disease.

## Discussion

IRIS is rare in patients after HSCT. Although IRIS was reported to be related to the discontinuation of immunosuppressive drugs (3, 4), in some cases, there was no significant correlation with this (5). Most patients developed IRIS within 3 months after HSCT (6, 7), but occasionally later, up to ~2 years after transplantation (5). In patients with SCID, the majority of BCG-IRIS appeared within 1 month after transplantation (8). In some cases, IRIS is accompanied by an increase in T cells (9). Searle et al. (10) reported BCG lymphadenitis related to the recovery of T cells occurring 83–164 days after HSCT counts in four patients who underwent HSCT due to familial hemophagocytic lymphohistiocytosis or malignant infantile osteopetrosis.

Our patient developed intermittent fever shortly after discontinued of CSA due to overdose. We diagnosed IRIS, as despite the increase in inflammation-related indicators, no new etiological evidence for his features was found, and treatment with antibiotics was ineffective. In addition, the effectiveness of anti-inflammatory treatment further confirmed the most likely the diagnosis of IRIS.

IRIS is divided into paradoxical IRIS and unmasking IRIS. Unmasking IRIS refers to the worsening of a pre-existing infection that has not been detected during the process of immune recovery, whereas paradoxical IRIS refers to the worsening of a confirmed pre-existing infection. Nine cases of paradoxical IRIS after HSCT were reported, and *Mycobacterium* was the most common pathogen (3, 4, 11, 12). We speculate that the IRIS of our patient was related to immune dysregulation following successful HSCT, including pre-existing BCG infection, immune reconstitution after HSCT, and withdrawal of immunosuppressants.

Not all IRIS requires treatment, and the majority of patients with IRIS have a good prognosis (5, 11–14). In addition to glucocorticoids and immunosuppressants, other treatment for IRIS includes non-steroidal anti-inflammatory drugs (NSAIDs) and immunosuppressants (11, 15, 16). Anti–IL-6 agent tocilizumab has also been used to treat IRIS due to elevated inflammatory factors (17). For our patient, because repeated episodes of high fever affected his daily life, methylprednisolone was initially administrated, but it was ineffective. Therefore, glucocorticoids and NSAIDs were administrated in combination, and with this regimen (and possibly the changed regimen of IVIG dose and frequency), the patient’s temperature returned to normal. However, because patients with IRIS have been reported with good prognosis without treatment, it cannot be ruled out that the reason for our patient’s recovery is only due to the passage of time. Although, we are not sure whether medications or time helped our patient’s recover. From the perspective of the correlation between drugs and the recovery of the patient, we still pointed out that the regimen with glucocorticoid and NSAIDs (and possibly IVIG) might have played a role.

In conclusion, we reported a case of BCG vaccine–related IRIS that occurred shortly after HSCT for SCID and after CSA withdrawal. Diagnosis of IRIS should be considered when immunocompromised patients present unexplained recurrent fever or worsening clinical manifestations of pre-existing infection after HSCT and related to immune reconstruction, when other possible etiology has been excluded.

## Data availability statement

The original contributions presented in the study are included in the article/supplementary material. Further inquiries can be directed to the corresponding author.

## Ethics statement

The studies involving human participants were reviewed and approved by Medical Ethics Committee, Beijing Children’s Hospital, Capital Medical University. Written informed consent to participate in this study was provided by the participants’ legal guardian/next of kin.

## Author contributions

All authors contributed to the patient’s care. SL, FH, and JW wrote the first draft of the manuscript. QW and HM contributed to the final version of the manuscript. All the authors contributed to the manuscript revision and read and approved the submitted version.

## Conflict of interest

The authors declare that the research was conducted in the absence of any commercial or financial relationships that could be construed as a potential conflict of interest.

## Publisher’s note

All claims expressed in this article are solely those of the authors and do not necessarily represent those of their affiliated organizations, or those of the publisher, the editors and the reviewers. Any product that may be evaluated in this article, or claim that may be made by its manufacturer, is not guaranteed or endorsed by the publisher.
